# Imhotep and the Discovery of Cerebrospinal Fluid

**DOI:** 10.1155/2014/256105

**Published:** 2014-03-13

**Authors:** Patric Blomstedt

**Affiliations:** Department of Pharmacology and Clinical Neuroscience, Umeå University, SE-901 85 Umeå, Sweden

## Abstract

Herbowski (2013) suggested recently the Egyptian Imhotep from the 3rd dynasty in Egypt to be the discoverer of cerebrospinal fluid. There are, however, no sources within the first 2000 years after Imhotep suggesting him to be in any way connected with the field of medicine. Over the course of three millennia Imhotep evolves into the sage who besides architecture also masters the arts of medicine, magic, astronomy, and astrology, at the same time as him being transformed from man to demi-God, and finally to a God. The identification of Imhotep as a doctor has thus little to do with facts and it is unlikely that he had anything to do with the Edwin-Smith papyrus from a much later period where CSF is first mentioned.

I read with interest the paper by Herbowski [1].

I would like to thank the author for his commendable desire to contribute to the history of this area. There is, however, an unfortunate assumption regarding the Egyptian origin of the discovery of cerebrospinal fluid (CSF). It is correct that the Edwin-Smith papyrus mentions what seems to be CSF, but the author takes this a step longer and attributes the discovery to Imhotep, writing “*there are enough necessary prerequisites to giving full credit to Imhotep for discovery of cerebrospinal fluid. Taking into consideration the content of The Papyrus of Smith, Imhotep can be fairly believed to be the very first discoverer of cerebrospinal fluid*,” and in the conclusions the author writes “*The Egyptian physician Imhotep is the most likely to be the first one to discover intracranial cerebrospinal fluid in vivo in 3000 B.C. The description of the discovery was found in The Papyrus of Smith of 1600 B.C.*” [1].

Herbowski is not the first enthusiastic doctor, with all likelihood not the last, to identify Imhotep as the origin behind the Edwin-Smith papyrus. Unfortunately, this identification is mostly based on the romantic allure of the idea itself and has very little to do with historical facts. The sources have been thoroughly evaluated by Sethe [2] and later in great detail by Wildung [3, 4].

We first meet the person Imhotep in the beginning of the Old Kingdom (27th century BC) as the chancellor of Djoser and supposed architect behind the first pyramid. There are only few, very short inscriptions regarding Imhotep that are close in time, and none of these suggests that he was a physician or had any medical knowledge [2].

The sources remain scarce for a long period, but he is mentioned as a wise man in a song from the New Kingdom, which is sometimes supposed to stem from the Middle Kingdom [5]. We have a reference to the chief lector-priest of Djoser, likely to be identical with Imhotep, in one of the stories in the Westcar papyrus [3, 6], dated to the Second Intermediate period. Later, in the 18th dynasty, he seems to have achieved a semidivine status, especially among scribes, and small statues of him as a scribe become popular. His rise to divinity and the first temples in his hounour occured around the 26th dynasty.

The divine field of Imhotep was not limited to medicine, but he was initially consulted by his adherers in a manner similar to other gods [2, 3]. The earliest health-related reference is as late as the 30th dynasty (4th century BC), where he appears as a divine healer [3]. However, over time the medical aspect becomes more and more pronounced and the Greeks identified him with Asclepius, and it seems likely that this identification with Asclepius did further enhance the medical aspect of Imhotep, and that cult reached its climax during the Roman period [3].

During Ptolemaic times we see the effects of an unclear delineation between the man and the god of medicine, and during later periods we meet the man Imhotep (even though often hardly identifiable with the original one) in many sources, for example, in the Greek Hermetic literature, where he now is identified as the first inventor of medicine [2, 7]. It might be added that over time his abilities in magic, alchemy, astronomy, and astrology also increase considerably [2, 3].

Today, the modern literature identifying Imhotep as a doctor is also highly influenced by a translation of Waddell of the Aegyptiaca, a history of Egypt in Greek, written two and a half millennia after Imhotep by the Egyptian priest Manetho in the late 3rd century BC [7, 8]. This work has been lost, but fragments have been preserved in other works from the 4th century AD and later [9]. Imhotep is, however, never mentioned in the writing of Manetho. According to the preserved fragments, Tosorthros, the second king of the 3rd dynasty (thus corresponding to Djoser), had the reputation of Asclepios among the Egyptians because of his medical skill. He was further the inventor of the art of building with hewn stone and devoted attention to writing. In modern times Maspero chose to translate Asclepios to Imhotep and identified the king with the God Imhotep. [10, 11]. Sethe suggested that since we know from other sources that Imhotep was identified by the Greeks with Asclepios, then this text might be referring to Imhotep, and not to the king [2]. This suggestion was accepted by Waddell in his translation of the fragments of Manetho in the Loeb edition [8], which is probably the most commonly consulted work regarding Manetho among nonscholars today. He simply inserted the name of Imhotep (Imuthês) in the text, as seen below.

**Tosorthros for 29 years. <In his reign lived Imuthês,> who because of his medical skill has the reputation of Asclepios among the Egyptians, and who was the inventor of the art of building with hewn stone. He also devoted attention to writing.**

It is today common to see the translation of Waddell, but without the signs marking Imhotep as an insertion/emendation of the translator. Considering what is mentioned of the medical skills of other kings, such as Athothis, this emendation is not acceptable (or as Wildung put it “So naheligend diese Emendierung ist, so unbewisbar und—so unötig is sie”). There is nothing suggesting that Imhotep was mentioned in the original text. The more modern translation of Adler has also rejected this emendation [9].

**Tosorthros, 29 years. Among the Egyptians, he is considered an Asclepius in recognition of his medical skill. The inventor of the art of building with hewn stone, he also persued the craft of writing.**

The insertion of the name of Imhotep into this passage in the 20th century AD and the reference to writing is the sole foundation of the statement that he might be the author of the first treatise on surgery, the Edwin-Smith papyrus. This suggestion was first made by Breasted [12], but a reading of his text gives the impression that he merely put this suggestion forward as a tantalizing idea.

Thus, in order to summarize, there are no sources within the first 2000 years after Imhotep suggesting him to be in any way connected with the field of medicine. Over the course of three millennia Imhotep evolves into the sage who besides architecture also masters the arts of medicine, magic, astronomy, and astrology, at the same time as his being transformed from man to demi-God, and finally to a God. How and why this development occurred are hidden in the mists of antiquity, but it should be obvious that the fact that a chancellor of Pharaoh was elevated to a God of medicine is not sufficient to state that he had any connection at all with medicine during his lifetime almost two millennia before this deification.

The search for the tomb of Imhotep has been pursued for a long time, and hopefully it will one day be found and settle the discussion concerning the role of Imhotep in the history of medicine. For now we can conclude with Estes that Imhotep had nothing to do with medicine “*at least, not before he became a god*” [13] and hence is unlikely to have anything to do with the Edwin-Smith papyrus or the discovery of CSF.
